# Transcriptional profiling of the host cell response to feline immunodeficiency virus infection

**DOI:** 10.1186/1743-422X-11-52

**Published:** 2014-03-19

**Authors:** Reinhard Ertl, Dieter Klein

**Affiliations:** 1 VetCore Facility for Research, University of Veterinary Medicine Vienna, Vienna, Austria

**Keywords:** Feline immunodeficiency virus, Host cell response, Virus-host interactions, Transcriptome sequencing

## Abstract

**Background:**

Feline immunodeficiency virus (FIV) is a widespread pathogen of the domestic cat and an important animal model for human immunodeficiency virus (HIV) research. In contrast to HIV, only limited information is available on the transcriptional host cell response to FIV infections. This study aims to identify FIV-induced gene expression changes in feline T-cells during the early phase of the infection. Illumina RNA-sequencing (RNA-seq) was used identify differentially expressed genes (DEGs) at 24 h after FIV infection.

**Results:**

After removal of low-quality reads, the remaining sequencing data were mapped against the cat genome and the numbers of mapping reads were counted for each gene. Regulated genes were identified through the comparison of FIV and mock-infected data sets. After statistical analysis and the removal of genes with insufficient coverage, we detected a total of 69 significantly DEGs (44 up- and 25 down-regulated genes) upon FIV infection. The results obtained by RNA-seq were validated by reverse transcription qPCR analysis for 10 genes.

**Discussion and conclusion:**

Out of the most distinct DEGs identified in this study, several genes are already known to interact with HIV in humans, indicating comparable effects of both viruses on the host cell gene expression and furthermore, highlighting the importance of FIV as a model system for HIV. In addition, a set of new genes not previously linked to virus infections could be identified. The provided list of virus-induced genes may represent useful information for future studies focusing on the molecular mechanisms of virus-host interactions in FIV pathogenesis.

## Background

Feline immunodeficiency virus (FIV) was first isolated from domestic cats in 1986 and has since then become an important animal model for human immunodeficiency virus (HIV) research [1,2]. Both viruses belong to the genus of lentiviruses and can be transmitted pre- and postnatally, via blood transfer and mucosal contact [2,3]. FIV can infect a broad range of cell types, including T-cells, B-cells, macrophages, and cells of the central nervous system. The genomes of FIV and HIV share the three main structural genes: *gag, pol* and *env*, as well as the two accessory genes: *rev* and *vif*. However, HIV encodes four additional accessory genes not present in FIV: *vpr, vpu, tat* and *nef*. In contrast, the *orf-A* gene can be found uniquely in FIV. Despite these differences on the genome level, FIV induced pathogeneses display striking similarities to human AIDS [2]. FIV progresses through three clinical stages, finally leading to an acquired immunodeficiency syndrome that increases the incidence of opportunistic infections and secondary diseases [4]. After the infection of a target cell, lentiviruses parasitize the cellular machinery to complete their life cycles. Following virus entry into the cell, the viral RNA genome is reverse transcribed and subsequently integrated into the cellular genome. The host cell machinery is then used to generate viral transcripts. These transcripts will be partially spliced and used as templates for the translation of the respective viral proteins. On the contrary, unspliced transcripts are incorporated into new virus particles, assembled from the structural viral proteins [5]. During all these steps, viral factors interact with multiple cellular proteins and hence, affect the normal course of cellular processes. These virus-induced changes of physiological processes can be detected on transcriptional and protein levels. For HIV, multiple cellular genes have been detected as differentially expressed at several stages of infection [6]. Some of these genes directly interact with viral proteins, whereas others might be only side-effects of the virus-induced changes in the host cell environment. Currently, only limited data is available about the impact of FIV infection on the host cell transcription. However, previous studies using cDNA microarrays suggest that transcriptional changes induced by FIV differ in between different cell types [7]. Furthermore, microarrays have been used to analyze the consequences of the viral Orf-A expression on the cellular mRNA profile [8]. In the present study, we use for the first time next-generation RNA sequencing (RNA-seq) to investigate the transcriptional host cell response to FIV [9]. T-cells were infected with FIV and the virus induced gene expression changes were investigated at 24 h post infection (hpi). The most significantly affected genes were additionally investigated by reverse transcriptase qPCR (RT-qPCR) at 8 and 24 hpi [10]. The results of this study will contribute gaining deeper insights into the complex network of virus-host interactions in FIV pathogenesis.

## Results

### Infection of T-cells and transcriptome sequencing

**Figure 1:**
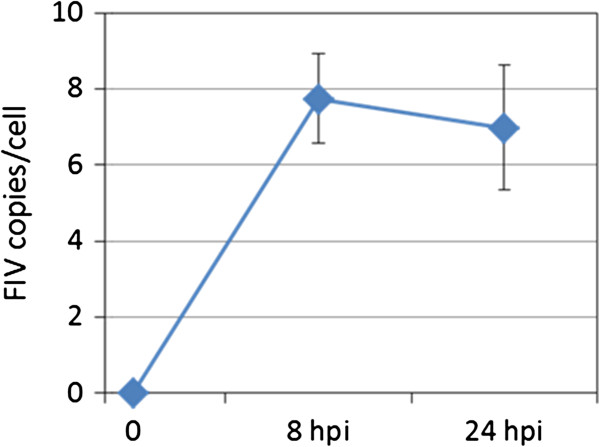
**Quantification of FIV provirus DNA in infected T-cells.** Numbers of FIV DNA copies per cell at 8 and 24 hpi were determined by qPCR.

FeT-J cells, a feline T-lymphocyte cell line, were infected with the FIV Petaluma strain. A high multiplicity of infection (MOI) of 10 virions per cell was used in order to ensure infection of the majority of target cells. FIV- and mock-infected cells were harvested at 8 and 24 hpi. Four replicates were analyzed for each time-point. The amounts of FIV provirus DNA were determined by quantitative PCR (qPCR) to estimate the infection efficiencies. At both time points similar provirus loads (7 FIV copies per cell) were detected, indicating successful infection of all replicate samples (Figure 1). The small decrease observed from 8 to 24 hpi can be explained by continuous cell division, while the production of new virus particles is expected to take a minimum of 24 h. High quality total RNAs (RNA integrity numbers of 10) of FIV (24 hpi) and mock infected cells were used for poly-A mRNA purification and the subsequent preparation of cDNA libraries for transcriptome sequencing. Next generation sequencing analysis on an Illumina platform generated a total of 42–57 million 37-bp reads per replicate sample (Table 1). Out of these, 40–56 million reads passed the quality filtering and were mapped against the cat reference genome. 60% of the filtered reads could be mapped to the cat genome. Additionally, in FIV infected cells 0.2% of reads were assigned to the FIV Petaluma genome suggesting the beginning of viral RNA transcription.

**Table 1:** Numbers of Illumina sequencing reads per replicate sample

**Sample**	**Description**	**Numbers of reads**			
		**Raw**	**Filtered**	**Mapping cat genome**	**Mapping FIV genome**
1	Mock	46,309,391	45,147,168	27,254,153	0
2	Mock	53,685,029	52,542,136	31,599,152	0
3	Mock	57,062,177	56,196,638	34,375,083	0
4	Mock	52,365,374	51,444,090	31,300,419	0
5	FIV 24 hpi	42,631,408	40,486,522	24,221,149	78,104
6	FIV 24 hpi	43,382,804	42,296,304	24,862,708	108,985
7	FIV 24 hpi	46,555,404	45,552,109	27,350,225	85,229
8	FIV 24 hpi	48,539,624	47,351,395	27,513,918	133,436

### Identification of differentially expressed genes

For quantification of transcript numbers, the filtered sequencing reads were assigned to the annotated genes present on the current version of the cat genome. Numbers of reads mapping to particular genes were counted and normalized by converting them into RPKM-values (reads per kilobase per million mapped reads) [11]. Out of 19,947 annotated genes present on the reference genome, sample reads could be assigned to 17,998 genes, leaving 1,949 genes without any mapping reads. For differential expression analysis, fold-changes and p-values were calculated in between the mean read counts of FIV-and mock-infected cells. The p-values were adjusted for multiple testing, according to the procedure proposed by Benjamini and Hochberg [12]. This statistical procedure was implemented to account for the multiple comparison problem that occurs during the analysis of high-throughput data. High numbers of hypotheses (e.g. differential expression of multiple genes) that are tested in one data set increase the probability that one of them is incorrectly accepted by chance. Correction methods, like Benjamin-Hochberg, are frequently used for large genomic data and call for adjustments, so that the probability of finding at least one significant result due to chance remains below the implemented significance level [12,13]. In order to ensure sufficient coverage of all analyzed genes, the threshold for transcription was set to a minimum RPKM-value of 0.5 in all replicate samples. Thus, genes with RPKM < 0.5 were excluded from the analysis. Genes exhibiting fold changes > 2 and adjusted p-values < 0.05 were considered as differentially expressed. This analysis strategy resulted in a total number of 69 differentially expressed genes (DEGs) at 24 h after FIV infection. The complete list of DEGs can be found in Additional file 1: Table S1. Out of these genes, 44 were up- and 25 down-regulated. The observed fold changes range from 2- to 5-fold, with the majority of genes being moderately (2- to 3-fold) up-regulated. Out of the 20 most significantly regulated genes, only 3 genes were down- and 17 were up-regulated (Table 2).

**Table 2:** List of top 20 most significantly regulated genes

**Gene symbol**	**Fold change**	**Log**_**2 **_**read counts**		**Adjusted p-value**	**Validation by RT-qPCR**
		**Mock**	**FIV 24 hpi**		
OASL	3.438	8.558	10.340	2.82E-07	Yes
ACHE	2.618	8.011	9.400	2.16E-06	Yes
DHX58	2.578	11.431	12.797	4.98E-06	No
DDX58	2.842	7.276	8.783	6.51E-06	No
TGM2	5.425	4.792	7.231	8.45E-06	Yes
HMGN2	-2.061	12.013	10.969	9.68E-05	Yes
CDKN1A	2.083	9.092	10.152	1.40E-04	Yes
ZFP36	2.040	7.806	8.834	2.98E-04	Yes
BCL6	2.102	3.744	4.817	3.32E-04	Yes
ASB2	5.465	3.050	5.500	3.72E-04	No
CSF1	2.042	7.056	8.087	3.91E-04	Yes
FITM1	2.082	2.227	3.286	4.69E-04	No
IFI44	2.587	4.470	5.841	1.75E-03	Yes
TP53INP1	2.226	4.988	6.143	1.77E-03	No
BMF	2.281	3.717	4.907	1.85E-03	No
SPINK4	-2.896	3.157	1.623	2.28E-03	No
HSPE1	2.985	3.005	4.583	3.20E-03	No
COA1	-2.516	3.559	2.227	3.54E-03	No
CXCL11	3.130	0.750	2.396	4.16E-03	Yes
ERBB2	2.312	1.123	2.333	5.42E-03	No

Most significantly regulated genes in FIV infected cells at 24 hpi as detected by RNA-seq. Fold changes were obtained by comparison of the mean read counts of FIV vs. mock infected cells. Genes were ranked according to the p-values.

### Validation of differential expressions by RT-qPCR

**Figure 2:**
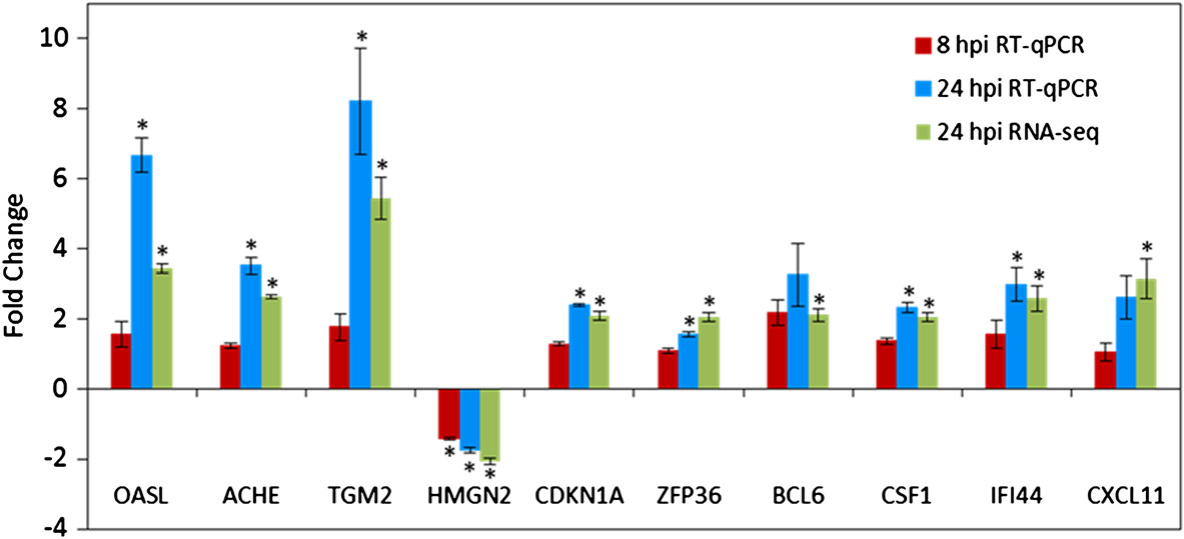
**Validation of 10 selected DEGs by RT-qPCR and comparison with RNA-sequencing.** RT-qPCR gene expression levels at 8 and 24 hpi were compared to mock infected cells. Fold changes were obtained by merging the mean values of 4 replicates for each time point, previously normalized to the expression of the 3 reference genes ABL, B2M and RSP7. Stars indicate significant (p < 0.05) expression changes.

RT-qPCR was performed in order to verify the results from transcriptome sequencing for 10 DEGs. The 10 genes were selected out of the 20 most significantly DEGs detected by RNA-seq (Table 2). Expression changes compared to mock-infected cells were additionally analyzed for an earlier time point, at 8 hpi. The FIV induced expression changes determined by RNA-seq could be confirmed by RT-qPCR for all investigated genes at 24 hpi (Figure 2). The highest virus induced changes were detected for TGM2 and OASL, analogue to the sequencing results. Compared to 24 hpi, the observed expression changes were generally lower at 8 hpi.

## Discussion

Since viruses have only a few genes, the use of the cellular machinery for the generation of viral transcripts and proteins is essential for their replication. Thus, virus replication interferes with normal cellular processes and therefore affects the expression of cellular genes. At present, only limited information is available on the effect of FIV infections on the host cell transcription. Here, we perform transcriptome analysis to investigate FIV induced gene expression changes in infected T-cells. A total of 69 DEGs could be identified at 24 hpi (Additional file 1: Table S1). As the validity of transcriptome data is dependent on sufficient numbers of sequencing reads mapping to all individual genes [14], we considered only genes with a minimum coverage (RPKM values > 0.5) for differential expression analysis. Due to this stringent threshold level, some lowly expressed genes were removed from the analysis. However, this approach could improve the reliability of the final list of DEGs. Furthermore, the expression patterns of 10 genes were confirmed by RT-qPCR (Figure 2), confirming that RNA-seq is a suitable method for the quantification of RNA expression levels. The comparison of RT-qPCR results for two time points (8 and 24 hpi) revealed generally lower expression changes for the 10 investigated genes at 8 hpi, thus indicating time-dependent induction of particular genes during the initial phase of infection. So far, only limited information is available about transcriptional changes modulated by FIV. However, previous studies investigated the impact of the viral *orf-A* gene on the host cell transcription. Here, alterations in several genes, responsible for post-transcriptional RNA modification and protein ubiquitination, were identified in T-cells expressing Orf-A, independently of the residual viral genes [8]. In contrast to that, we could not detect changes for these genes in our data. We assume that these discrepancies can be mainly attributed to different experimental setups, especially regarding the exposure time to the stimulus, as we investigated the early antiviral response whereas the previous study used stable Orf-A expressing cell lines [8]. Moreover, these results may also indicate differences in the cellular responses to individual viral proteins compared to complete viruses.

### FIV induced gene expression changes: Similarities to HIV

Out of the 69 FIV induced genes (Additional file 1: Table S1); several genes have already been described to be affected also by infection with other viruses, in particular HIV. OASL, the most significantly regulated gene upon FIV infection (up-regulated 3.4-fold; Table 2) is known as an antiviral protein, induced by a variety of viruses, such as the influenza A and hepatitis C virus [15,16]. Other examples include DHX58 and DDX58 (up 2.6- and 2.8-folds), belonging to the group of RNA helicases. These enzymes are involved in many processes of the cellular RNA metabolism including the sensing of viral RNAs and the mediation of antiviral immune responses [17]. ACHE (up 2.6-fold) encodes for an enzyme necessary for the termination of neuro-transmitted signals. Furthermore, the plasma secretion of this protein is accomplished via exosome-like vesicles that are also used by HIV for the release of viral Nef proteins. Thus, HIV increases ACHE expression and vesicle production in order to enhance the export of its own proteins [18]. We detected TGM2 to be highly up-regulated (up 5.4-fold) after FIV infection, similar to previous findings for HIV [19]. This multifunctional protein is involved in several cellular processes including apoptosis. The induction of TGM2 might be an antiviral mechanism of the host cells in order to trigger apoptosis and interfere with virus replication [20]. HMGN2 (down 2.1-fold) is presumed to be involved in HIV replication due to interactions with the viral integrase [21]. CDKN1A (other names: p21/waf1; up 2.1-fold) encodes a cyclin-dependent kinase inhibitor that functions as a regulator of cell cycle progression, especially in the promotion of cell cycle arrest. HIV affects the function of the encoded protein and thus, influences cell cycle progression [22]. The induction of cell cycle arrest might benefit viruses because of the resulting suppression of immune responses and the generation of an optimized cellular environment for viral reproduction [23]. In contrast, the increase of ZFP36 (tristetraprolin; up 2.0-fold) might be an antiviral response of the host cell. ZFP36, due to its zinc finger motifs, is able to interact with specific AU-rich regions of HIV RNAs and consequently targets them for rapid degradation [24]. Together with other cellular defense mechanisms, this process may represent an additional antiviral barrier that aims to block virus replication at the transcription level. Similar functions, as a transcriptional repressor of HIV have also been described for BCL6 (up 2.1-fold) [25]. Other examples of genes affected by FIV that have previously been associated with HIV pathogenesis include CSF1, IFI44, HSPE1 and CXCL11. The protein encoded by CSF1 (up 2.0-fold) is a secreted cytokine that controls the production, differentiation and function of macrophages. The induction of this pro-survival cytokine by HIV facilitates protection infected cells from apoptosis [26]. IFI44 (MTAp44; up 2.6-fold) is inducible by interferons (IFNs) which are released by cells in response to the presence of viruses, bacteria and parasites. IFNs trigger the early antiviral defense of the immune system that aims to eradicate the pathogens. IFI44 is frequently upregulated in virus infected cells generating an antiproliferative state, impeding viral replication [27,28]. HSPE1 (up 3.0-fold) encodes a major heat shock protein. These stress proteins are expressed in response to a broad range of stimuli such as heat and microbial infections. Additionally, interactions with HIV suggest functions in the regulation of viral gene expression and replication [29]. Another IFN induced gene is CXCL11 (up 3.1-fold) which functions as a chemokine and is the dominant ligand for the CXC receptor-3 on immune cells. Its release initiates a chemotactic response in activated T-cells recruiting them to lymphoid organs. In the case of lentiviral infections, the migration of additional cells to infected lymph nodes increases the numbers of potential target cells and thus, contributes to the spread of infection [30].

Several of the DEGs identified in this study are known to interact with HIV accessory proteins that are missing in FIV. The HIV Tat protein functions as an activator of viral RNA transcription and is responsible for the induction of several cellular genes, such as CDKN1A [31], BCL6 [32], IFI44 [27], C1QTNF5, EEFCC1 [33] and COX4I1 [34]. For CDKN1A, additional interactions have been described with HIV Vpr [35]. As we observed expression changes for all these genes also in this study, similar mechanisms can be assumed for FIV. Here, other viral genes might adopt the particular functions attributed to HIV Tat and Vpr.

## Conclusion

Taken together, we investigated the virus induced gene expression changes in FIV infected T-cells. Using next-generation sequencing technology, a total of 69 DEGs were identified at 24 hpi. Out of those genes most significantly affected by FIV, several genes could be associated to known interactions with HIV in humans. These findings indicate a similar effect of both viruses on the host cell transcription and further underline the importance of FIV as a model system for HIV research. The virus-induced genes identified in this study represent potential cellular factors involved in FIV pathogenesis and may serve as a basis for future investigations aiming to provide new insights into the molecular mechanisms of lentiviral infections.

## Materials and methods

### Cells and viruses

FeT-J cells were purchased from ATCC (CRL-11967). Cells were cultivated in RPMI 1640 medium supplemented with 10% FBS, 1 mM sodium pyruvate, 10 mM HEPES and 0.05 mM 2-mercaptoethanol. All media and supplements were purchased from PAA Laboratories (Pasching, Austria). A molecular clone of the FIV Petaluma strain was propagated in FeT-J cells [36]. Parts of the virus genome, *gag* and *orf-A* genes (Genbank: JF411740, JF411742) have been sequenced for previous studies [37]. Herewith, we detected 99% sequence homology with the wild-type Petaluma isolate (Genbank: M25381).

### Virus preparation and quantification

Supernatants from FIV-infected FeT-J cells were harvested by centrifugation and 0.45 μm filtered. For virus quantification, the amount of FIV RNA in the supernatant was measured by RT-qPCR. Therefore, viral RNA was isolated and DNase I digested using the Direct-zol RNA Miniprep Kit (Zymo Research, Irvine, USA) and used as a template for RT-qPCR analysis with FIV *gag* specific primers and a TaqMan probe [38]. Primer sequences can be found in Additional file 2: Table S2. FIV RNA copy numbers were estimated relative to a tenfold dilution series of an *in-vitro* transcribed RNA standard. PCR reactions conditions and standard generation have been described earlier [38]. The obtained FIV RNA copy numbers were then converted into virions/ml supernatant. Virus preparations were stored at -80°C until further use for infection experiments.

### Infection

A spinoculation protocol was performed in order to increase the numbers of virus particles binding to the target cells [39]. Briefly, FeT-J cells were resuspended in FIV-containing supernatant at a MOI of 10 and a cell density of 1 × 10^6^ cells/ml. Cells were then seeded into 6-well plates and centrifuged at 1200 g for 2 h at 25°C. Afterwards, the cells were washed three times with cold medium, transferred into new culture plates and cultivated for 8 and 24 h. Control cells were mock-infected with FIV-free supernatant and treated in the same way. Four replicate samples were infected for each time point. The harvested cells were split in half and either frozen at -20°C for DNA isolation or immediately resuspended in Trizol reagent (Life Technologies, Carlsbad, USA) for subsequent RNA isolation.

### Quantification of FIV DNA

FIV provirus DNA in infected cells was estimated by qPCR in order to quantify the infection rates. DNA was isolated utilizing a commercial kit (DNeasy Blood and Tissue Kit, Qiagen, Hilden, Germany) according to the manufacturers’ instructions. qPCR analysis was performed with TaqMan probe assays targeting the FIV *gag*[38] and the cat RPP30 gene, 30 kDa subunit of ribonuclease P (Additional file 2: Table S2). The RPP30 sequence was used for a blast search against the cat genome, locating one hit on chromosome D2. Thus, analog to the human genome [40], we assume that there are two allelic copies RPP30 per cell in the cat genome and used this qPCR assay for the quantification of cell numbers (2 copies RPP30 = 1 cell). For the generation of plasmid DNA standards, parts of the FIV *gag* and RPP30 gene were amplified by PCR and cloned into the pCR2.1 plasmid using the TOPO TA cloning Kit (Invitrogen, Carlsbad, USA). The prepared plasmid DNA was used for the generation of standard dilutions ranging from 1 × 10° – 1 × 10^6^ copies FIV or RPP30. Relating to both standards, FIV and RPP30 copy numbers were determined and converted into FIV copies/cell. Reaction conditions for the TaqMan probe qPCR have been described earlier [37].

### Transcriptome sequencing

Cellular RNA was isolated using the Direct-zol RNA Miniprep Kit (Zymo Research) including an on-column DNase I digestion for the removal of gDNA. RNA quality control was performed by capillary electrophoretic separation of the samples on the Agilent 2100 Bioanalyzer (Agilent Technologies, Santa Clara, USA) and subsequent estimation of RNA integrity numbers. 1 μg of total RNA were used as the starting material for the preparation of cDNA libraries utilizing the TruSeq RNA Sample Prep Kit v2 (Illumina, San Diego, USA) following the recommended protocol. Sequencing was performed on a Genome Analyzer IIx instrument (Illumina) with reagents taken from the TruSeq SR Cluster Kit v5 and TruSeq SBS Kit v5 (Illumina). The lengths of the generated sequencing reads were 37 bp. The raw sequencing data are available in the ArrayExpress database [http://www.ebi.ac.uk/arrayexpress] under accession number E-MTAB-2083.

### Sequencing data analysis

The Trimmomatic filtering tool was used for quality control and trimming of the sequencing reads [41]. The data were scanned for contamination with adapter sequences from library preparation. Additionally, all bases below an Illumina quality score of 25 and reads shorter than 25 bp were removed. The trimmed data set was then mapped against the cat genome release 72 from Ensembl database [ftp://ftp.ensembl.org/pub/release-72/genbank/felis_catus/] and the FIV Petaluma genome (Genbank: M25381) using the QSeq v5 analysis software (DNASTAR, Madison, USA) implementing the default options for Illumina short reads (matching of a minimum of 20 bases and at least 80% of bases within each read). The numbers of raw reads mapping to annotated genes on the genome reference were counted and fold changes were calculated by comparing the mean values of four FIV infected samples to four control samples. P-values (Student’s t-test) were calculated in QSeq v5 and adjusted according to the Benjamini-Hochberg method [12]. Genes were considered to be differentially expressed; exhibiting fold changes > 2, adjusted p-values < 0.05 and RPKM expression values greater than 0.5 in all replicate samples [11]. For those genes, which were specified as ‘novel’ or ‘uncharacterized’ in the present version of the cat genome, a blast search in NCBI database was performed and the homologous human gene symbols were used for further analysis.

### RT-qPCR analysis of gene expression

Total RNA was converted into cDNA using the High Capacity Reverse Transcription Kit (Life Technologies) following the manufacturer’s instructions. Primers were designed using the Primer Express 3.0 software (Life Technologies) and validated by the determination of PCR reaction efficiencies as previously described [42]. All primers are described in Additional file 2: Table S2. RT-qPCR was performed in 20 μl reactions including 2 μl cDNA template, 200 nM of each primer, 0.2 mM of each dNTP, 3 mM MgCl_2_, 1 x Solution S, 1 x buffer B2, 0.4 x EvaGreen fluorescent dye (Biotium, Hayward, USA) and 1 unit of HOT FIREPol DNA polymerase. All reagents for RT-qPCR were purchased from Solis BioDyne (Tartu, Estonia). Reactions were run on a Viia 7 real-time PCR instrument (Life Technologies) using the following temperature protocol: 95°C for 10 min, 45 cycles of 95°C for 15 sec and 60°C for 1 min, followed by the generation of melting curves. Target gene expression levels were normalized to those of three references genes, namely ABL, B2M and RSP7 [43]. Relative expression changes of FIV- vs. mock-infected cells were estimated using the comparative 2^^-∆∆CT^ method [44].

### Data deposition

The RNA-sequencing reads reported in this paper have been deposited in the ArrayExpress database [http://www.ebi.ac.uk/arrayexpress] under accession number E-MTAB-2083.

## Abbreviations

DEGs: Differentially expressed genes; FIV: Feline immunodeficiency virus; HIV: Human immunodeficiency virus; hpi: Hours post infection; IFN: Interferon; MOI: Multiplicity of infection; qPCR: Quantitative PCR; RNA-seq: RNA-sequencing; RPKM: Reads per kilobase per million mapped reads; RT-qPCR: Reverse transcription quantitative PCR.

## Competing interest

The authors declare that they have no competing interests.

## Authors’ contributions

RE and DK conceived and designed the experiments. RE performed the experiments, analyzed the data and wrote the manuscript. All authors edited and approved the final manuscript.

## Supplementary Material

Additional file 1: Table S1 List of differentially expressed genes in FIV infected T-cells at 24 hpi as detected by RNA-seq analysis. Fold changes refer to mock-infected cells. Read counts (log_2_) represent the mean values of four replicate samples. P-values were adjusted according to the Benjamini-Hochberg method. Click here for file

Additional file 2: Table S2 Primers used for quantitative PCR. Click here for file
