# Supplementary material for: Transcriptional profiling of the host cell response to feline immunodeficiency virus infection
Source: Virol J. 2014 Mar 19;11:52. doi: 10.1186/1743-422X-11-52 (PMC3999937; doi:10.1186/1743-422X-11-52)
Supplement: Additional file 2: Table S2 — Primers used for quantitative PCR. [file 1743-422X-11-52-S2.pdf]

Name	Oligo	Sequence 5’ - 3‘	Accession number	PCR efficiency	Reference
FIV gag	Forward	GCAGAAGCAAGATTTGCACCA	M25381	1.021	[38]
	Reverse	TATGGCGGCCAATTTTCCT			
	Probe	FAM-TGCCTCAAGATACCATGCTCTACACTGCA-BHQ1			
RPP30	Forward	GCCAAGTGTGAGGGCTGAAA	XM_003994205	1.048	-
	Reverse	GTGGACGGACTACCAAATGGA			
	Probe	HEX-CCAGTCCTCGTCAGCGCTCCCTTC-BHQ1			
OASL	Forward	GTGAAACATCGGCCAACCA	ENSFCAT00000009643	0.950	-
	Reverse	TGGCTTTCACGTACTGCAGGTA			
ACHE	Forward	GTGCTCGGGTCTATGCCTACA	ENSFCAG00000023546	1.001	-
	Reverse	GCCCAAAGATGAACTCGATCTC			
TGM2	Forward	CCCGAGTGGTGACCAACTATAAC	ENSFCAT00000004809	1.029	-
	Reverse	GCGGAAGTACTCGATGAGCAA			
HMGN2	Forward	AAGCTGATGCTGGCAAGGA	ENSFCAT00000032519	0.964	-
	Reverse	TCTGTGCCTGGTCTGTTTTGG			
CDKN1A	Forward	GGCAGACCAGCATGACAGATT	ENSFCAT00000025177	0.987	-
	Reverse	CAGATTAGGGCTTCCTCTTGGA			
ZFP36	Forward	CCACCCCAAGTACAAGACAGAACT	ENSFCAT00000031857	0.939	-
	Reverse	GATGCTCTGGCGAAGCACAT			
BCL6	Forward	CGTGATGGCTACGGCTATGTAC	ENSFCAT00000006812	0.931	-
	Reverse	CGGCTTCACTGGCCTTGATA			
CSF1	Forward	GATGGAGACTTCGTGCCAAATT	ENSFCAT00000010774	0.923	-
	Reverse	AGGTAGCACACGGGATCTTTCA			
IFI44	Forward	ACACGTACAGGACATATTCCATTAAGG	ENSFCAT00000027884	0.951	-
	Reverse	CTCATGCAGGCCCATTGTG			
CXCL11	Forward	ATTGTTCAAGGTTTCCCCATGTT	ENSFCAT00000026314	1.032	-
	Reverse	GCTTTCTCAATATCTGCCACTTTCA			
ABL	Forward	TGTGGCGAGTGGTGATAATACAC	ENSFCAT00000005306	0.985	[43]
	Reverse	TCCACTCACCATTCTGGTTGTAA			
B2M	Forward	CGCGTTTTGTGGTCTTGGT	NM_001009876	0.953	[43]
	Reverse	AAACCTGAACCTTTGGAGAATGC			
RSP7	Forward	GTCCCAGAAGCCGCACTTT	NM_001009832	0.991	[43]
	Reverse	CACAATCTCGCTCGGGAAAA			

Additional file 2
